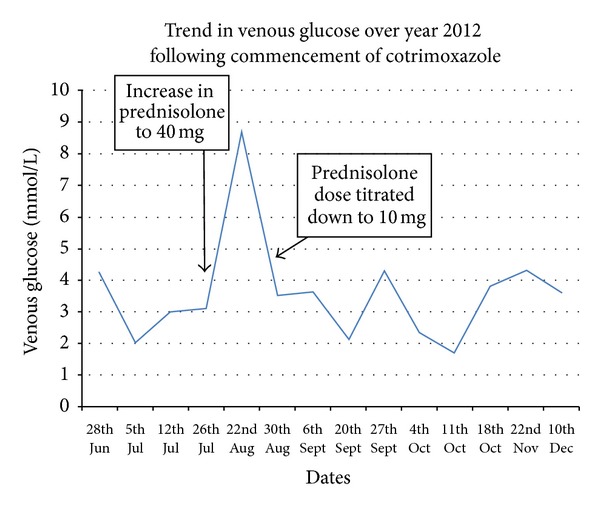# Cotrimoxazole-Induced Hypoglycaemia in a Patient with Churg-Strauss Syndrome

**DOI:** 10.1155/2013/415810

**Published:** 2013-09-08

**Authors:** Russell Senanayake, Mamoun Mukhtar

**Affiliations:** ^1^Royal Berkshire NHS Foundation Trust, Reading RG1 5AN, UK; ^2^Diabetes and Endocrinology Department, Melrose House, Royal Berkshire Hospital, Reading RG1 5AN, UK

## Abstract

Cotrimoxazole is a commonly used antimicrobial agent which is traditionally indicated in the management of pneumocystis infection of which HIV and immunosuppressed individuals are at high risk. Furthermore, it can be used on the long term for prophylactic indications. Hypoglycaemia following commencement of cotrimaoxazole is a rare adverse effect which was first described in 1988. We describe a case of hypoglycaemia shortly following initiation of cotrimoxazole indicated as long-term prophylaxis on a background of Churg-Strauss syndrome. The patient was symptomatic for hypoglycaemia despite simultaneous use of high-dose prednisolone; however, the hypoglycaemia did not require a hospital admission. We will explore the risk factors, monitoring requirements, and the mechanism by which co-trimoxazole induces hypoglycaemia.

## 1. Introduction

Cotrimoxazole (combination of trimethoprim and sulfamethoxazole) is an antimicrobial agent which has both prophylactic and therapeutic indications for* Pneumocystis jirovecii *infection which represents a high risk among immunosuppressed patients. Pneumocystis is an opportunistic infection which often presents as fever, nonproductive cough, and dyspnoea in HIV patients although symptoms are not as marked and with a relatively shorter duration in immunocompromised patients for reasons other than HIV [1]. It is typically identified on microscopy which can be obtained from bronchoscopy with bronchoalveolar lavage. Mortality associated with pneumocystis infection has been linked to mechanical ventilation needs or failure in antimicrobial treatment [2]. 

Cotrimoxazole is commonly used as long term prophylaxis in HIV-infected patients to promote increased survival [3]. Furthermore, it has been trialed with positive results in the management of drug-resistant superbugs [4]. The efficacy of cotrimoxazole has made it ideal as first line management in both treatment and prophylaxis of pneumocystis infection. Despite this, there are high rates of adverse effects and potential for P. jirovecii drug resistance [5]. Alternative chemoprophylactic agents for pneumocystis infections include pentamidine and dapsone. The most common adverse effect associated with cotrimoxazole is gastric discomfort and reduced appetite. 

We discuss a case of cotrimoxazole-induced hypoglycaemia initiated in a patient with Churg-Strauss syndrome. Of the case reports we analysed detailing hypoglycaemia secondary to cotrimoxazole in our discussion section the patients were either on high dose cotrimoxazole or were otherwise elderly frail and had renal impairment or were severely immunocompromised as a direct consequence of a comorbidity. Our case interestingly describes a patient who was immunocompromised as a consequence of a short course of high dose steroid therapy; however; she was otherwise young and fit. Furthermore, our case outlines how the patient had symptomatic Cotrimoxazole-induced hypoglycaemia despite concurrent use of high dose steroid therapy. 

## 2. A Case Presentation

A 52-year-old lady was referred to the endocrinology department following development of dizziness, intense hunger, headaches, and excessive sweating. Symptoms onset coincided with onset of fleeting pulmonary infiltrates, >20% eosinophilia, long standing sinusitis, nasal polyps, and mononeuritis multiplex associated with a new diagnosis of Churg-Strauss syndrome. The patient's initial medication on diagnosis included prednisolone 40 mg OD, azathioprine 100 mg OD, omeprazole 20 mg OD, and cotrimoxazole 480 mg OD which was started in June 2012 as prophylaxis. She was also on a 4-month course of Cyclophosphamide which was commenced on diagnosis. The patient reported worsening dizziness and excessive sweating following a decrease in prednisolone dose in early July with symptoms amelioration on increasing prednisolone to 40 mg during a clinic review after a month. Despite this, the patient experienced neither severe hypoglycaemic features such as collapse and fits nor impact on driving.

Her baseline investigations including full blood count and renal and liver functions were all normal. A review of weekly nonfasting venous glucose concentrations from July demonstrated a range of 2–4.5 mmol/L with the lowest recording being 1.7 mmol/L. This trend in venous glucose relative to concurrent prednisolone dosing is summarised in Figure 1. Both the insulin levels (296 pmol/L) and C-peptide levels (2031 pmol/L) were inappropriately raised. The patient took glucose oral supplements up to four times daily to mediate temporary symptom relief. At no point did the patient require a hospital nor intensive care admission with intravenous glucose supplementation.

The patient was advised to discontinue the cotrimoxazole after three months following which there was a resolution in hypoglycaemic symptoms with no further requirement for daily oral glucose supplementation. A decision was made for the patient not to be restarted on cotrimoxazole.

## 3. Discussion

We describe a case symptomatic cotrimoxazole-induced hypoglycaemia with associated low venous glucose readings. Hypoglycaemia associated with initiation of cotrimoxazole was first described in 1988 in an HIV patient treated for pneumocystis [6]. Our patient had developed a pattern of daily oral glucose self-administration to ameliorate symptoms including headache and dizziness. Fortunately, the patient did not require hospital admission even at the point where venous glucose recording was 1.7 mmol/L. With the case studies, we reviewed that cotrimoxazole-induced hypoglycaemia would require several intravenous glucose boluses to maintain normoglycaemic ranges [7–9]. The amelioration of the hypoglycaemia symptoms preventing a hospital admission in our patient may have been achieved by the simultaneous daily administration of high dose prednisolone which is not present in the case studies we reviewed. The potentially severe sequelae implicated by cotrimoxazole may suggest the need for glucose monitoring in patients on long term cotrimoxazole. Despite this, a review of the available literature highlights the rarity of cotrimoxazole induced hypoglycaemia which suggests that routine glucose monitoring in such patients would not be considered cost-effective. Nevertheless, it is important for clinicians to be vigilant of emerging hypoglycaemic features during the review of patients on long term cotrimoxazole. 

Cotrimoxazole holds many structural similarities to sulphonylureas [10]. The sulfa component of cotrimoxazole appears to be responsible for the hypoglycaemic adverse effect. It has been postulated that it binds to insulin *β* cells and creates a state of insulin hypersecretion. This theory is supported by the role of prednisolone which was simultaneously prescribed in our patient. Prednisolone influences glucose metabolism by promoting insulin resistance of peripheral glucose-dependent tissues. This insulin resistance may partly antagonise the insulin hypersecretion derived from the sulfamethoxazole component of cotrimoxazole. We experienced a progressive fall in venous glucose results as the prednisolone dose was titrated down. There were additional findings of elevated serum insulin and C-peptide levels in our patient which further supports cotrimoxazole's role in causing insulin hypersecretion. One review identifies that the serum insulin levels were raised in 88% of the 14 cases evaluated with 28% cases demonstrating elevated C-peptide [7]. 

Studies have identified that the trimethoprim component selectively inhibits CYP2C8 and sulfamethoxazole inhibits CYP2C9 [11]. It therefore can promote hypoglycaemia with a sulphonylurea by inhibiting hepatic metabolism of sulphonylureas. Screening of our patient's liver function shortly following admission failed to identify liver function derangement which may impair cotrimoxazole metabolism. Cotrimoxazole has additionally been identified to enhance the action of repaglinide by its selective inhibitory action on CYP2C8 [8]. This highlights the importance of careful cotrimoxazole prescribing in the setting of simultaneous oral hypoglycaemic agents. Our patient was not on any oral hypoglycaemic agents although she was on omeprazole. Omeprazole may be implicated in promoting hypoglycaemia; [12] however, this appears to be in combination with *H. pylori* triple drug therapy and is not a recognised adverse effect of long term isolated omeprazole use. 

Our patient had preserved renal function with eGFR > 60 at the point of diagnosis with Churg-Strauss. Renal impairment is not a prominent feature observed in Churg-Strauss; however, the prevalence rates are highly variable [13]. Impaired renal function is recognized as a risk factor for hypoglycaemia [14]. An estimated 10% to 30% of cotrimoxazole is renally excreted [15]; however, the larger proportion undergoes hepatic excretion. It is therefore important to evaluate the patient's baseline renal and hepatic functions before commencing cotrimoxazole. It should be prescribed with caution in patients with chronic kidney disease stage 4 and end-stage renal failure. The key learning points in this case are outlined as follows.

*Learning Points. *When prescribing Cotrimoxazole, consider the following.

1. Evaluate the baseline renal and hepatic function before commencements.
2. Be vigilant of evolving hypoglycaemic features during the review of patients.
3. Use with caution in patients simultaneously taking oral hypoglycaemic agents, particularly Sulphonylureas.

## Figures and Tables

**Figure 1:**